# Comment on Leivaditis et al. Between Air and Artery: A History of Cardiopulmonary Bypass and the Rise of Modern Cardiac Surgery. *J. Cardiovasc. Dev. Dis.* 2025, *12*, 365

**DOI:** 10.3390/jcdd12100409

**Published:** 2025-10-16

**Authors:** Khaled Ebrahim Al Ebrahim

**Affiliations:** Surgery Department, King Abdulaziz University, Jeddah 21589, Saudi Arabia; dr.k.ebrahim@gmail.com; Tel.: +966-591532286

**Keywords:** cardiopulmonary bypass, cardiac surgery, surgical innovation, history of medicine, Ibn al-Nafīs, Islamic medicine

We read with great interest the historical review by Leivaditis et al. on the evolution of cardiopulmonary bypass (CPB) and its transformative impact on modern cardiac surgery [1]. The authors provide a comprehensive account of the intellectual and technological milestones that culminated in the development of the heart–lung machine. It cannot be overstated that the rise of cardiac surgery in the 1950s was made possible by this brilliant and ingenious invention. Since then, millions of procedures have been performed worldwide, and CPB has become the cornerstone of modern cardiac surgery.

We would like to highlight an important historical contribution that deserves greater emphasis: the role of Arab and Islamic scholars, particularly Ibn al-Nafīs (1210–1288). In his Commentary on Avicenna’s Canon (c.1242), Ibn al-Nafīs was the first to provide a correct description of pulmonary circulation. He rejected Galen’s theory of invisible pores in the interventricular septum, stating that blood cannot pass directly from the right to the left ventricle. Instead, he described how blood is propelled from the right ventricle to the lungs via the pulmonary artery, mixes with air in the spongy pulmonary tissue, and then returns to the left ventricle through the pulmonary vein [2]. This was a profound break from the Galenic dogma and a pivotal step toward the modern understanding of cardiopulmonary physiology, centuries before William Harvey’s description of systemic circulation. While Ibn al-Nafīs stands out as a pioneer, other Arab and Muslim scholars have also contributed to cardiovascular knowledge. Avicenna (Ibn Sīnā, 980–1037) in his influential Canon of Medicine has provided the detailed accounts of the pulse, cardiac anatomy, and physiology, synthesizing and transmitting classical knowledge that dominated medical teaching for centuries. Ibn Rushd (Averroes, 1126–1198) and Ibn Zuhr (Avenzoar, 1091–1161) preserved and critiqued the Galenic thought, helping to establish the intellectual environment in which Ibn al-Nafīs could challenge long-held errors [3]. These contributions reflect the rich and multicultural heritage of medical science that ultimately paved the way for breakthroughs like CPB. Over the past seven decades, CPB has undergone continuous refinement to minimize complications and improve outcomes. Advances such as biocompatible circuits, miniaturized extracorporeal circulation (MiECC) systems, membrane oxygenators, and artificial intelligence-assisted perfusion have all contributed to safer and more effective procedures. Despite the development of off-pump cardiac surgery and modern percutaneous interventional procedures, CPB remains indispensable in the future of cardiac surgery [4]. Its versatility and safety in complex congenital, valvular, and ischemic heart disease makes it the cornerstone of cardiac surgery. As CPB approaches its 70th anniversary, it continues to exemplify the extraordinary synergy of engineering, physiology, and surgical innovation.
